# A Combination of Acetate, Propionate, and Butyrate Increases Glucose Uptake in C2C12 Myotubes

**DOI:** 10.3390/nu15040946

**Published:** 2023-02-14

**Authors:** Britt M. J. Otten, Mireille M. J. P. E. Sthijns, Freddy J. Troost

**Affiliations:** Food Innovation and Health, Department of Human Biology, Faculty of Health, Medicine and Life Sciences, School of Nutrition and Translational Research in Metabolism (NUTRIM), Maastricht University, 5928 SZ Venlo, The Netherlands

**Keywords:** C2C12 myotubes, short chain fatty acids, glucose uptake, glutathione

## Abstract

Background: Dietary fibers are subjected to saccharolytic fermentation by the gut microbiota, leading to the production of short chain fatty acids (SCFAs). SCFAs act as signaling molecules to different cells in the human body including skeletal muscle cells. The ability of SCFAs to induce multiple signaling pathways, involving nuclear erythroid 2-related factor 2 (Nrf2), may contribute to the redox balance, and thereby may be involved in glucose homeostasis. The aim of this study is to investigate whether SCFAs increase glucose uptake by upregulating the endogenous antioxidant glutathione (GSH) in C2C12 myotubes. Methods: C2C12 myotubes were exposed to 1, 5, or 20 mM of single (acetate, propionate, or butyrate) or mixtures of SCFAs for 24 h. Cytotoxicity, glucose uptake, and intracellular GSH levels were measured. Results: 20 mM of mixture but not separate SCFAs induced cytotoxicity. Exposure to a mixture of SCFAs at 5 mM increased glucose uptake in myotubes, while 20 mM of propionate, butyrate, and mixtures decreased glucose uptake. Exposure to single SCFAs increased GSH levels in myotubes; however, SCFAs did not prevent the menadione-induced decrease in glucose uptake in myotubes. Conclusions: The effect of SCFAs on modulating glucose uptake in myotubes is not associated with the effect on endogenous GSH levels.

## 1. Introduction

The gut microbiota plays an important role in skeletal muscle function and consequently in enhancing physical exercise performance [1,2]. Metabolites derived from microbial fermentation play an important role in the microbes–host metabolic crosstalk [3]. Saccharolytic fermentation of dietary fibers in the proximal colon by the gut microbiota leads to the production of favorable metabolites such as short chain fatty acids (SCFAs) [4]. The main SCFAs formed by the gut bacteria are acetate (C2), propionate (C3), and butyrate (C4) [5]. The molar ratio between acetate:propionate:butyrate in the large intestine is approximately 60:20:20 [6]. SCFAs are largely taken up by colonocytes, where butyrate is the main energy source [7]. SCFAs that are not metabolized in colonocytes are transported to the liver where a large part of propionate and butyrate is taken up. Acetate uptake in the liver is low, which results in the highest plasma concentration compared to the other SCFAs. The SCFAs that are not processed by the liver can be metabolized by other tissues, such as skeletal muscle [8]. The molar ratio between acetate:propionate:butyrate in peripheral blood of healthy subjects is approximately 80:10:10 [6].

Short chain fatty acids can affect skeletal muscle function in various ways including binding on membrane-bound G-protein couple receptors (GPR) 41 or GPR43, intracellularly by active transport, or passive diffusion into the cell [9,10]. Binding of SCFAs to GPR41 and GPR43 activates several intracellular pathways [11]. The pathways activated by these receptors include release of intracellular Ca^2+^, ERK1/2 activation, and inhibition of cAMP accumulation [11,12]. Although both GPRs are activated by SCFAs, they have different specificity and physiological function. Propionate is the most potent agonist for both GPR41 and GPR43 and acetate was selective for GPR43, whereas butyrate was active for GPR41. Through passive diffusion butyrate has been shown to inhibit histone deacetylase (HDAC). HDAC inhibitors have shown potent anti-inflammatory activity in inflammatory diseases [13].

During exercise the production of reactive oxygen species (ROS) within skeletal muscle increases [13]. Low levels of ROS that are generated during exercise promote many signaling pathways that are involved in skeletal muscle metabolism, mitochondrial biogenesis, and mitochondrial function, as well as antioxidant enzymes that regulate intracellular ROS levels [14]. These adaptations of skeletal muscle cell may lead to resistance against oxidative damage via antioxidant pathways. ROS induced activation of 5′-adenosine monophosphate-activated protein kinase (AMPK) activates the peroxisome proliferator-activated-receptor-gamma-coactivator-1*α*-(PGC-1*α*) signal transduction pathway [15]. This is important in regulating mitochondrial biogenesis and function in a PGC-1*α*-dependent pathway and stimulates glucose transporter 4 (GLUT4) translocation to the plasma membrane, and a concomitant increase in glucose transport [15,16]. 

The intracellular antioxidant capacity plays an important role in maintaining ROS levels in a physiologically compatible range. This allows ROS to serve as a signaling molecule while preventing too high ROS levels which may exert direct toxic effects [17]. However, when the cell is exposed to excessive ROS for a long period of time or in large concentrations relative to the endogenous antioxidant levels, the redox balance cannot be maintained and this may result in oxidative damage to DNA, lipids, and proteins [18]. Every cell has multiple endogenous antioxidant systems including the GSH system, thioredoxin system, different vitamins, and protective enzymes such as catalase or superoxide dismutase that can be upregulated to restore this balance. Nuclear factor erythroid 2-related factor 2 (Nrf2), a regulator of cellular antioxidant defenses [19,20], is the primary transcription factor protecting cells from oxidative stress by regulating the antioxidant GSH pathway [21]. The SCFAs propionate and butyrate have been shown to increase Nrf2 nuclear translocation in various cell types, e.g., hepatocytes and endothelial cells [22]. Through this mechanism of action SCFAs have shown to protect against oxidative stress and inflammation in diabetic mice [23].

Despite the positive effect of SCFAs on skeletal muscle metabolism and function it is questioned whether they could increase glucose uptake by regulation of the antioxidant system in skeletal muscle. Therefore, the aim of this study is to investigate if SCFAs increase glucose uptake by upregulating the endogenous antioxidant GSH in myotubes. It is hypothesized that acetate, propionate, and butyrate separately and combined in physiological ratios increase GSH, which results in increased glucose uptake in myotubes. To investigate this, a C2C12 murine muscle cell line was used. Here, we show that a mixture of SCFAs increases glucose uptake in myotubes while the observed effect is not associated with changes in endogenous GSH levels.

## 2. Materials and Methods

### 2.1. Chemicals

Acetic acid, propionic acid and butyric acid were purchased from Sigma-Aldrich (Saint Louis, MO, USA) as well as bovine serum albumin fatty-acid free (BSA), human insulin solution, 4-(2-hydroxyethyl)-1-piperazineethanesulfonic acid (HEPES), Triton X-100 (TX-100), β-nicotinamide adenine dinucleotide reduced disodium salt hydrate (NADH), sodium pyruvate, β-nicotinamide adenine dinucleotide 2′-phosphate reduced tetrasodium salt hydrate (NADPH), sulfosalicylic acid solution (SSA), Ethylenediaminetetraacetic acid tetrasodium salt dihydrate (EDTA), reduced glutathione (GSH), oxidized glutathione (GSSG), glutathione reductase (GR), 2-vinylpyridine (2-VP), 5,5ʹ-Dithio-*bis*-(2-nitrobenzoic Acid) (DTNB), Potassium dihydrogen phosphate (KH_2_PO_4_), Dipotassium hydrogen phosphate (K_2_HPO_4_), human insulin solution, metformin, and menadione. Ethanol and the Pierce bicinchoninic acid (BCA) protein assay kit were purchased from ThermoFisher Scientific (Fremont, CA, USA). The Glucose Uptake-Glo assay kit was purchased from Promega (Leiden, The Netherlands).

### 2.2. Cell Culture and Treatments

A C2C12 murine myoblast cell line (ATCC; CRL-1772; Manassas, VA, USA) was cultured at passage 9–16 in growth medium (GM), composed of Dulbecco’s Modified Eagle medium (DMEM, Gibco, Carlsbad, CA, USA) supplemented with 9% (*v*/*v*) fetal bovine serum (FBS, Gibco), and 1% (*v*/*v*) antibiotics (100 ug/mL penicillin, 100 µg/mL streptomycin, Gibco). Cells were cultured in a humidified atmosphere containing 5% CO_2_ at 37 °C until 70–80% confluency was reached.

**Figure 1:**
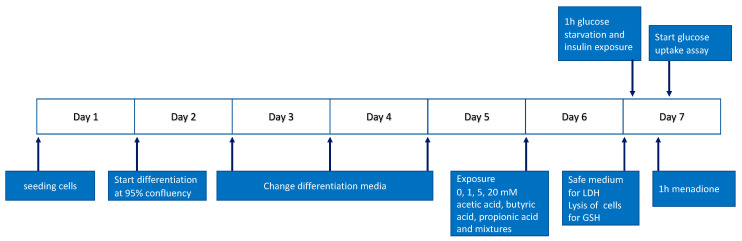
Experimental timeline.

Depending on the experiment, C2C12 myoblasts were seeded on Matrigel coated (Corning Life sciences, Corning, NY, USA) 6-well or 24-well culture plates (Greiner Bio-One, Frickenhausen, Germany) (Figure 1) at a density of 1 × 10^4^ cells/cm^2^ and cultured in GM for 1.5 days until reaching ±95% confluency. After reaching ±95% confluency, cells were washed with Dulbecco’s phosphate-buffered saline (PBS, Gibco) and differentiation medium (DM) was added, which was composed of 1% (*v*/*v*) heat inactivated FBS (hiFBS, Gibco) and 2.5% (*v*/*v*) HEPES. DM was refreshed every day for 3 consecutive days. After 4 days of differentiation, cells were incubated for 24 h with a 3% bovine serum albumin (BSA, Sigma-Aldrich) solution containing either single (acetate, propionate, or butyrate) or mixtures of SCFAs in concentrations of 1, 5, or 20 mM (Table 1).

### 2.3. Cytotoxicity Measurement

Cytotoxicity was assessed by measuring the presence of the cytosolic enzyme lactate dehydrogenase (LDH) in the medium. A 3% TX-100 media solution was used to induce cell lysis, which was assumed to induce 100% LDH release and served as a positive control. A 100 mM pyruvate potassium phosphate solution with 377 mM NADH was added. All LDH levels were expressed as a percentage of LDH activity as seen after cell lysis and was shown relative to the condition in which the cells were not exposed to SCFA. NADH absorption was measured at λ = 340 nm with a microplate reader every 20 s for 4 min at 37 °C and the slope of the curve was calculated.

### 2.4. Glucose Uptake Measurement

2-deoxyglucose (2-DG) uptake into C2C12 myotubes was assessed using the Glucose Uptake-Glo assay kit according to the manufacturer’s instructions. Briefly, C2C12 myoblasts were differentiated into myotubes on a 24-wells plate and were exposed to single- and mixtures of SCFAs for 24 h (Figure 1). Metformin was added to the C2C12 myotubes at a concentration of 400 µM for 24 h as a positive control. After 24 h, cells were glucose starved for 1 h and exposed to 100 nM insulin for 1 h before incubation with 2-DG (100 µM, 30 min). The reaction was stopped by adding an acid detergent stop solution. Samples were transferred to a 96 non-transparent luminescent plate and a pH neutralization solution was added. In total, 100 µL of a detection reagent containing glucose-6-phosphate dehydrogenase (G6PDH), NADP+, reductase, luciferase, and luciferin substrate was added, and luminescence was measured at a 0.3–1 s integration using a microplate reader. Changes in glucose uptake were expressed as fold change compared to controls without SCFAs and insulin exposure.

### 2.5. GSH Measurement

To determine the amount of GSH in C2C12 myotubes, cells were lysed using a 100 mM potassium phosphate solution (pH 7,5) supplemented with 10 mM EDTA containing 1% TX-100 and incubated on ice for 30 min. Cells were scraped and transferred to a 2 mL Eppendorf tube. All lysates were centrifuged at 14,000× *g* for 10 min at 4 °C to remove cell debris. Total protein content of the supernatant was determined with a bicinchoninic acid assay (BCA) (ThermoFisher, Boston, MA, USA). In addition, 300 µL of the supernatant was mixed with 300 µL 6% (*v*/*v*) SSA in milliQ water. To determine the GSH concentration, Rahman’s enzymatic recycling method was used as previously described [24]. The absorption of TNB was measured at λ = 412 nm for 10 min at 37 °C and the slope of the curve was calculated. A calibration curve of GSH and GSSG was made to determine the concentrations of GSH and GSSG over time. GSH was calculated by subtraction of GSSG from total GSH. All samples were corrected for the amount of protein measured.

### 2.6. Statistical Analysis

All values were presented as the mean ± SEM. Normality was checked with the Shapiro–Wilk test. In case of statistical significance of this test, the nonparametric Mann–Whitney test was used to test significant difference between two individual conditions. When normally distributed, a two-tailed independent sample *t*-test was used. At least three independent experiments were performed in duplicates as well as the analyses. Statistical tests were performed using GraphPad Prism 9.4 software (GraphPad, Prism, La Jolla, CA, USA). *p*-values < 0.05 were considered statistically significant.

## 3. Results

### 3.1. Mixture 1, a Combination of SCFAs, Is Cytotoxic to C2C12 Myotubes at a Concentration of 20 mM

**Figure 2:**
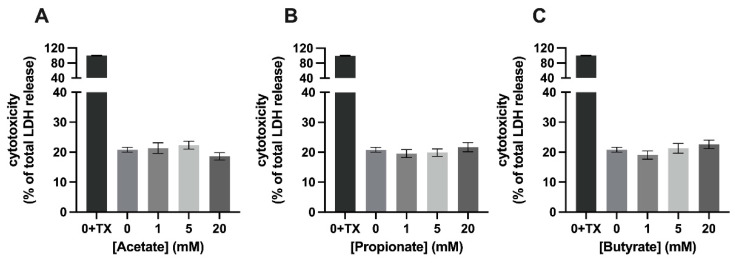
**Cytotoxicity in C2C12 myotubes exposed to single SCFAs.** Concentrations of 1, 5, and 20 mM of acetate (**A**), propionate (**B**), and butyrate (**C**) exposed for 24 h were not cytotoxic to C2C12 myotubes. Cytotoxicity is shown as a percentage of LDH activity seen after complete cell lysis (addition of 3% TX-100) and expressed relative to cytotoxicity shown at exposure to 0 mM (*N* ≥ 5; *n* = 2). All data are presented as mean ± SEM.

**Figure 3:**
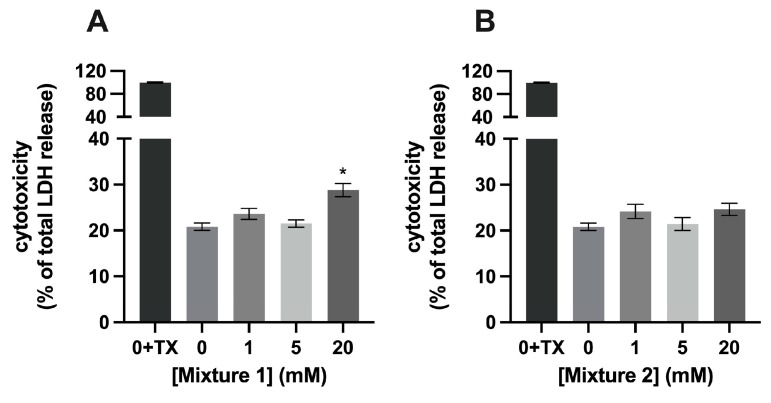
**Cytotoxicity in C2C12 myotubes exposed to different mixtures of SCFAs.** (**A**) A concentration of 20 mM of mixture 1 exposed for 24 h on C2C12 myotubes increased cytotoxic compared to 0 mM SCFA. (**B**) Mixture 2 was not cytotoxic to C2C12 myotubes at concentrations of 0–20 mM exposed for 24 h. Cytotoxicity is shown as a percentage of LDH activity seen after complete cell lysis (addition of 3% TX-100) and expressed relative to cytotoxicity shown at exposure to 0 mM (*N* = 3; *n* = 2). All data are presented as mean ± SEM. * *p* < 0.001 compared to 0 mM SCFA exposure.

Exposure of C2C12 myotubes to 1, 5, or 20 mM of single SCFAs for 24 h was not cytotoxic compared to control (=0 mM SCFA) (Figure 2). However, after 24 h of exposure of the C2C12 myotubes to 20 mM of mixture 1, LDH release increased by 8.0 % (*p* < 0.001) compared to control (20.82 ± 0.8 % and 28.8 ± 1.4% LDH, respectively) (Figure 3A), while exposing of the myotubes to 1, 5, or 20 mM of mixture 2 for 24 h did not induce changes in LDH release and, hence, in cytotoxicity compared to control (Figure 3B).

### 3.2. Exposure to 5 mM of Mixture 1 Increased Glucose Uptake in C2C12 Myotubes

**Figure 4:**
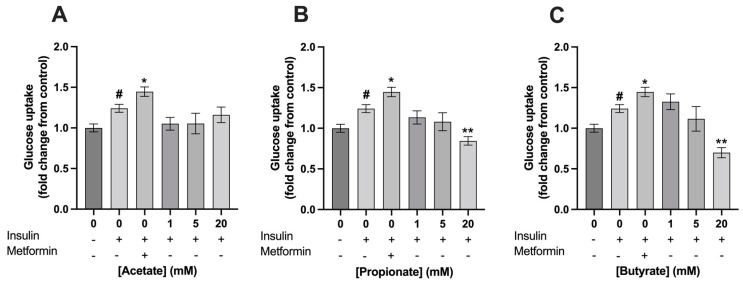
**Glucose uptake in C2C12 myotubes exposed to single SCFAs did not increase glucose uptake.** Acetate did not induce any effects (**A**), while 20 mM propionate and 20 mM butyrate significantly decreased glucose uptake in C2C12 myotubes (**B**,**C**). Metformin (400 µM, 24 h) increased glucose uptake in C2C12 myotubes (*N* ≥ 3, *n* = 2). All data are presented as mean ± SEM. # *p* < 0.01 compared to 0 mM SCFA, * *p* < 0.01 compared to 0 mM SCFA treated with 100 nM insulin; ** *p* < 0.001 compared to 0 mM SCFA treated with 100 nM insulin. Fold change was calculated compared to control without insulin treatment.

**Figure 5:**
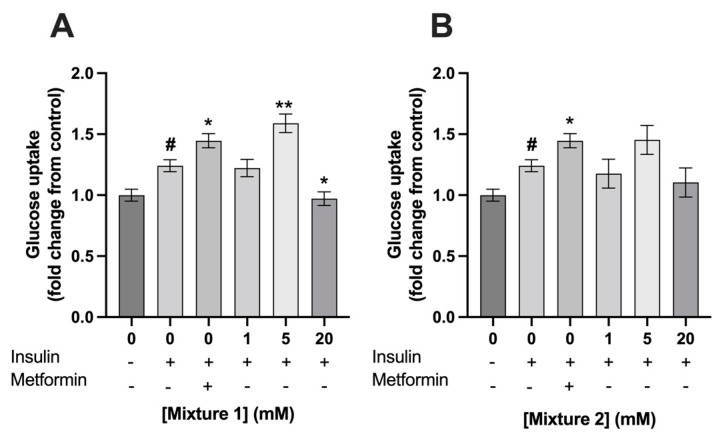
**Exposure to 5 mM mixture 1 of SCFAs stimulated glucose uptake in C2C12 myotubes.** A 24 h exposure to mixture 1 at 5 mM increased glucose uptake in C2C12 myotubes (**A**). In addition, a similar trend was seen for mixture 2 (**B**). Metformin (400 µM, 24 h) increased glucose uptake in C2C12 myotubes (*N* ≥ 3, *n* = 2). All data are presented as mean ± SEM. # *p* < 0.01 compared to 0 mM SCFA * *p* < 0.01 compared to 0 mM SCFA treated with 100 nM insulin; ** *p* < 0.001 compared to 0 mM SCFA treated with 100 nM insulin. Fold change was calculated compared to control without insulin treatment.

The exposure of C2C12 myotubes to 100 nM insulin increased its glucose uptake by a fold change of 1.2 ± 0.2 compared to control (*p* < 0.001). Exposure to 400 µM metformin for 24 h, which was used as a positive control, increased glucose uptake in C2C12 myotubes with a fold change of 1.4 ± 0.06. Metformin significantly increased insulin dependent glucose uptake compared to 0 mM SCFA, which was only treated with insulin (1.4 ± 0.06 vs. 1.2 ± 0.05, respectively; *p* < 0.01) (Figure 4 and Figure 5)). Acetate had no effect on insulin dependent glucose uptake compared to 0 mM SCFA, which was only treated with insulin (*p* > 0.05) (Figure 4A), while propionate and butyrate, at a concentration of 20 mM, decreased insulin dependent glucose uptake in C2C12 with a fold change of 0.8 ± 0.07 and 0.7 ± 0.06 (*p* < 0.01 and *p* < 0.001), respectively (Figure 4B,C). Mixture 1, at a concentration of 20 mM, decreased insulin dependent glucose uptake as well 0.97 ± 0.07 (*p* = 0.03) (Figure 5A). In contrast, a 24 h exposure of 5 mM of mixture 1 significantly increased insulin dependent glucose uptake in C2C12 myotubes with a fold change of 1.6 ± 0.09 compared to control (*p* < 0.01) (Figure 5A). A similar increasing trend was seen for 5 mM of mixture 2 with a fold change of 1.5 ± 0.1 compared to control, however, this did not reach statistically significance (*p* = 0.06) (Figure 5B).

### 3.3. Exposure to SCFAs Increased GSH Levels in C2C12 Myotubes

**Figure 6:**
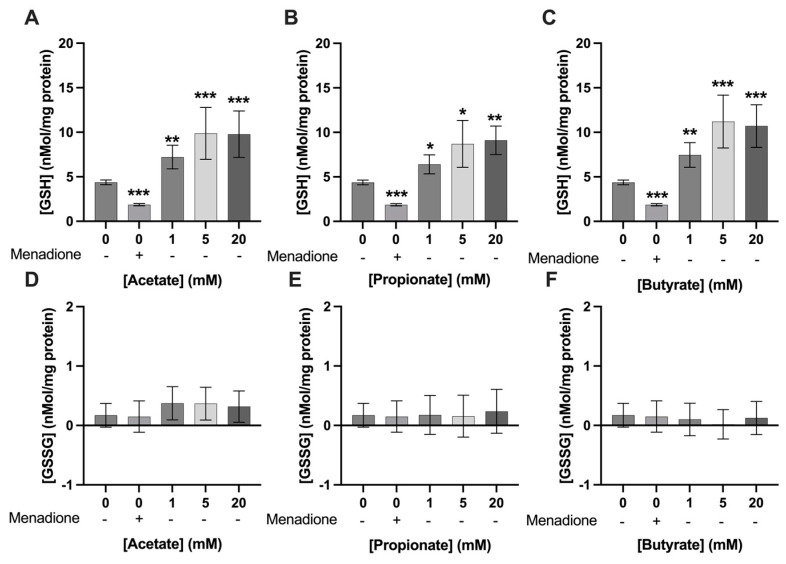
**Single SCFAs increased GSH levels in C2C12 myotubes.** Menadione (25 µM) significantly decreased GSH levels in C2C12 myotubes. The SCFAs acetate (**A**), propionate (**B**), and butyrate (**C**) at concentrations ranging from 1–20 mM significantly increased GSH levels in C2C12 myotubes. Menadione, acetate (**D**), propionate (**E**), nor butyrate (**F**) altered GSSG levels significantly compared to control (*N* ≥ 4; *n* = 2). All data are presented as mean ± SEM. * *p* < 0.05; ** *p* < 0.01; *** *p* < 0.001 compared to 0 mM exposure.

**Figure 7:**
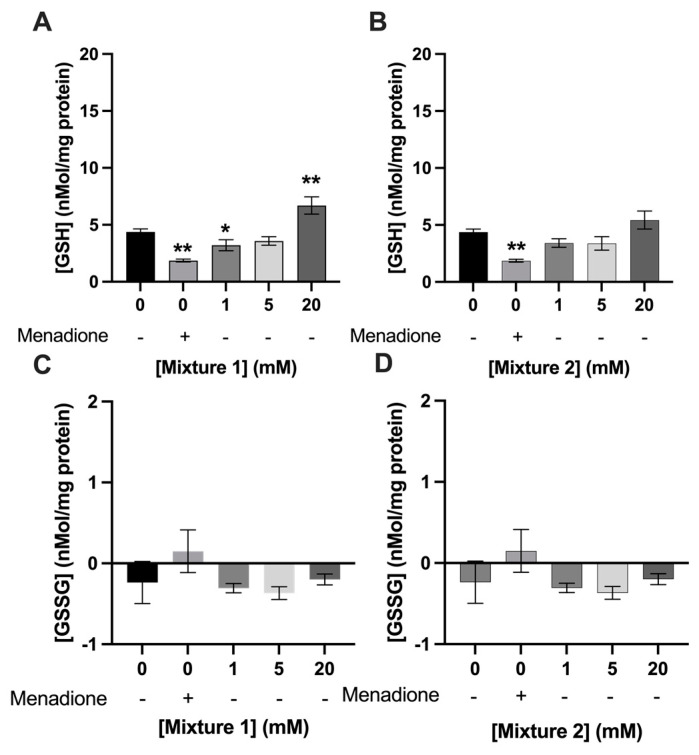
**20 mM of mixture 1 increased GSH levels while 1 mM decreased GSH in C2C12 myotubes.** Menadione (25 µM) significantly decreased GSH levels in C2C12 myotubes. Twenty four hours of exposure to mixture 1 at 20 mM increased GSH levels in C2C12 myotubes (**A**), while 1 mM decreased GSH levels compared to 0 mM exposure. Mixture 2 did not change GSH or GSSG levels in C2C12 myotubes (**B**). Menadione, mixture 1 (**C**), nor mixture 2 (**D**) altered GSSG levels significantly compared to control (*N* ≥ 3, *n* = 2). All data are presented as mean ± SEM. * *p* < 0.05; ** *p* < 0.001 compared to 0 mM exposure.

A concentration of 25 µM of menadione significantly decreased GSH levels in C2C12 myotubes compared to 0 mM exposure (1.9 ± 0.1 nmol/mg protein vs 4.4 ± 0.3 nmol/mg protein; respectively, *p* < 0.01) (Figure 6 and Figure 7). Acetate, butyrate, and propionate, at 1, 5, and 20 mM, increased GSH levels in C2C12 myotubes compared to control (Figure 6A–C). Menadione and SCFAs did not change GSSG levels significantly compared to control (*p* > 0.05) (Figure 6D–F). Twenty mM of mixture 1 increased GSH levels in C2C12 myotubes compared to 0 mM exposure (6.7 ± 0.8 nmol/mg protein 4.4 ± 0.3 nmol/mg protein; respectively, *p* < 0.001) (Figure 7A), whereas mixture 2 did not significantly change GSH levels compared to control (*p* > 0.05) (Figure 7B). Both mixture 1 and 2 did not change GSSG levels compared to 0 mM SCFA exposure (Figure 7C,D).

### 3.4. Single- and Mixtures of SCFAs Did Not Prevent the Menadione-Induced Decrease in Glucose Uptake in C2C12 Myotubes

**Figure 8:**
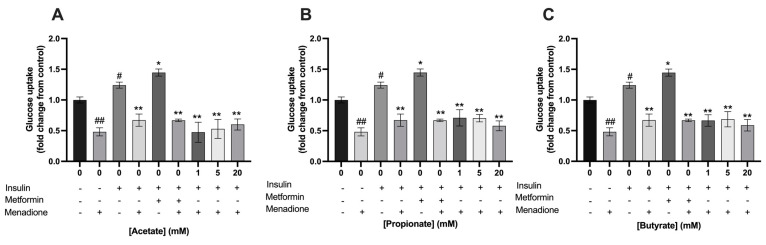
**Exposure to single SCFAs did not prevent menadione induced reduction in glucose uptake in C2C12 myotubes**. Exposure to acetate (**A**), propionate (**B**), nor butyrate (**C**) prevented menadione-induced reduction in glucose uptake in C2C12 myotubes (*N* ≥ 3, *n* = 2). All data are presented as mean ± SEM. # *p* < 0.01 compared to 0 mM SCFA, ## *p* < 0.001 compared to 0 mM SCFA, * *p* < 0.01 compared to 0 mM SCFA treated with 100 nM insulin; ** *p* < 0.001 compared to 0 mM SCFA treated with 100 nM insulin. Fold change was calculated from control without insulin treatment.

**Figure 9:**
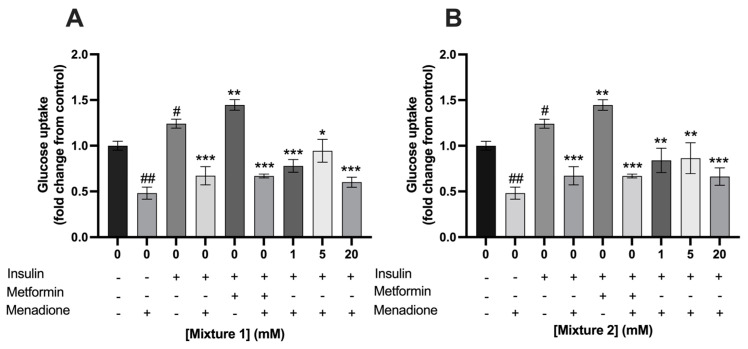
**Exposure to a mixture of SCFAs did not prevent menadione induced reduction in glucose uptake in C2C12 myotubes**. Exposure to mixture 1 (**A**) or mixture 2 (**B**) did not prevent menadione-induced reduction in glucose uptake in C2C12 myotubes (*N* ≥ 3, *n* = 2). All data are presented as mean ± SEM. # *p* < 0.01 compared to 0 mM SCFA, ## *p* < 0.001 compared to 0 mM SCFA, * *p* < 0.05 compared to 0 mM SCFA treated with 100 nM insulin; ** *p* < 0.01 compared to 0 mM SCFA treated with 100 nM insulin; *** *p* < 0.001 compared to 0 mM SCFA treated with 100 nM insulin. Fold change was calculated from control without insulin treatment.

Menadione at a concentration of 25 µM, to induce oxidative stress, significantly decreased glucose uptake in C2C12 myotubes compared to control in the absence of insulin (0.48 ± 0.07 vs. 1.0 ± 0.05; *p* < 0.001, respectively) (Figure 8 and Figure 9). The addition of acetate, propionate, or butyrate separately or in a mixture did not prevent glucose uptake after 1 h of menadione exposure compared to control in the presence of insulin.

## 4. Discussion

We showed that a mixture of acetate, propionate, and butyrate in relative ratios of 60:20:20, at a concentration of 5 mM induced an increase in glucose uptake in C2C12 myotubes. Single SCFAs did not increase glucose uptake, while high concentrations of 20 mM propionate and butyrate even decreased glucose uptake in myotubes. Single SCFAs increased GSH levels in myotubes while the mixtures of SCFAs did not increase GSH levels. In conclusion, the effects of SCFAs on glucose uptake in myoblasts were not associated with its’ effects on endogenous GSH levels.

Skeletal muscle is thought to account for 70 to 90% of insulin-stimulated glucose storage [25]. Results from mouse C2C12 and rat L6 muscle cell lines rarely demonstrate an increased fold change of 2-fold increase in glucose uptake with maximum insulin exposure [26]. These results are in line with findings from this study, in which we showed that exposure of C2C12 cells to insulin induced an increase in glucose uptake of 1.2-fold. A maximum increase in glucose uptake by a 1.6-fold change was shown by a combination of the SCFAs acetate, propionate, and butyrate in relative ratio 60:20:20. A similar trend was seen for mixture 2, in which the SCFAs were given in the ratio 80:10:10. Interestingly, the combined but not the single SCFAs increased glucose uptake significantly. Remarkably, the increase in glucose uptake induced by mixture 1 was even higher compared to metformin-induced increase in glucose uptake. Metformin increases GLUT4-mediated glucose uptake through an insulin-independent signaling pathway targeting AMPK activation and subsequently enhance GLUT4 translocation to the plasma membrane [27]. AMPK is a key regulator for maintaining homeostasis in energy metabolism [28]. It was previously established that SCFAs are able to phosphorylate AMPK in myotubes and skeletal muscle [29,30,31], likely by increasing the expression of PPAR-δ. Butyrate has been shown to increase the expression of PPAR-δ in both L6 myotubes and skeletal muscle in C57BL/6J mice in vivo [29]. However, Hernandez et al. (2021) showed no increase in AMPK phosphorylation following acute or chronic acetate treatment (0 up to 5 mM exposure) in human skeletal muscle cells [32]. Another mechanism of SCFAs is the inhibition of HDACs, which can be induced by passive diffusion of SCFAs into the cell. HDACs possess key roles in maintaining skeletal muscle metabolic homeostasis, regulating skeletal muscles adaptation and exercise capacity [33]. The inhibition of HDACs may also play a key role in insulin sensitivity as increased GLUT4 translocation and basal and insulin-induced glucose uptake in skeletal muscle is observed after the inhibition of HDAC in L6 myotubes [34]. This leads to an increase in insulin receptor substrate 1 (IRS1) expression and protein kinase B (PKB) phosphorylation.

Another binding target of SCFAs on skeletal muscle cells are the transmembrane G protein-coupled receptors (GPRs) that are activated by SCFAs and induce intracellular signaling cascades and cellular responses [35]. More specifically, GPR41 and GPR43, also known as free fatty acid receptor 3 (FFAR3) and FFAR2, respectively, are the best identified and studied SCFA receptors present on the skeletal muscle [36,37]. Activation of both receptors results in Gα subunit disassociates from the Gβγ subunits and couples with Gα_i/o_ proteins which inhibits the activity of adenylate cyclase (AC) and leads to reduced generation of cyclic adenosine monophosphate (cAMP) [11]. In addition, activation of GPR43 also activates phospholipase C (PLC) via Gα_q/11_ and promotes activation of inositol triphosphate (IP3) receptors on the endoplasmic reticulum (ER) [37]. This results in G*α*_q/11_-induced increase in Ca^2+^ release into the cytoplasm [38]. This effect was demonstrated in L6 myotubes which were exposed to acetate [39]. The effect of acetate on intracellular Ca^2+^ influx was inhibited in L6 myotube that were knocked down in GPR43 by transfection of GPR43 specific siRNA. Lahiri et al. (2019) exposed differentiated C2C12 cells to a 10 mM cocktail of SCFAs, in a similar ratio as mixture 1 (60:25:15; acetate: propionate: butyrate, respectively) and observed a significant increase in PGC-1α [40]. PGC-1α is a transcriptional coactivator that is a central inducer of mitochondrial biogenesis in cells as it can also modulate the composition and functions of individual mitochondria [41]. Although in this study relative ratios of 60:20:20 showed to improve glucose uptake, it is unknown what the effect is on mitochondrial biogenesis, as well as on the oxidative capacity. Furthermore, the contribution of each specific GPR receptor on mitochondrial biogenesis, as well as the oxidative capacity is still unknown.

Skeletal muscle glucose uptake as well as mitochondrial activation are essential for the energy homeostasis. Increased energy generation is essential for muscle contraction. In addition, SCFA binding to GPR41 and GPR43 activation leads to Ca^2+^ release from the ER which is also important in skeletal muscle contraction [42]. Furthermore, Ca^2+^ also regulates intracellular processes, such as myosin–actin cross bridging, protein synthesis, protein degradation and fiber type shifting by the control of Ca^2+^-sensitive proteases and transcription factors, as well as mitochondrial adaptations and respiration [42]. Butyrate-induced AMPK phosphorylation increases levels of PGC-1*α* in insulin-resistant hepatocytes and in mice, resulting in increased skeletal muscle glucose uptake and an increase in insulin sensitivity [43]. The affinity ranking for activation of GPR43 by different SCFAs is ordered as acetate = propionate > butyrate [11]. In contrast, for GPR41 the preferred order is propionate > butyrate > acetate. Due to the difference in binding affination for the SCFAs on the GPR41/43 insulin dependent may explain why the separate SCFAs did not increase glucose uptake, whereas in mixture 1 they seem to induce a synergistic effect on the glucose uptake. An amount of 20 mM of propionate, butyrate, and mixture 1 decreased the glucose uptake, which could be linked to an increase in cytotoxicity.

This study shows that intracellular glutathione levels in C2C12 myotubes increase after exposure to the single SCFAs; however, this did not prevent an oxidative stress-induced decrease in glucose uptake. This indicates that the glutathione synthesis pathway is activated separately from the glucose uptake pathway. In addition, 20 mM of propionate, butyrate, and mixture 1 increased GSH levels but were associated with a decrease in glucose levels. Furthermore, 20 mM of mixture 1 showed an increase in LDH release in the medium indicating cytotoxicity. Compared to 1 and 5 mM exposure of the single and mixtures of SCFAs, high levels of GSH are observed in 20 mM. This may indicate increased stress levels in the cells as stressed cells and oxidative stress itself also induce Nrf2 [44].

In the present study we showed that a specific combination of SCFAs increased glucose uptake in C2C12 myotubes. These results suggest that colonic fermentation of dietary fibers, which results in the production of different SCFAs in a specific ratio, has the potential to increase skeletal muscle glucose uptake. This may be beneficial for nutritional management of health and disease, i.e., in athletes or other people who have a need to take up more glucose by skeletal muscle. Future research should include other physiologically relevant ratios of SCFAs and identify the underlying mechanisms. This will eventually need to culminate in human clinical trials in which targeted SCFA production by gut microbiota will be modified by nutrition intervention, such as with prebiotics, to assess the in vivo implication of the currently presented results on glucose uptake. In addition, the contribution of the SCFAs on both GPR41 and GPR43 activation and its signaling pathways remains to be investigated. Furthermore, it would be of interest to investigate if our findings on increased GSH levels also apply to other endogenous antioxidant systems that are regulated by Nrf2.

In conclusion, exposure to the SCFAs acetate, propionate, or butyrate, was not associated with increased glucose uptake in C2C12 myotubes. A combination of these SCFAs in relative ratio 60:20:20 increased glucose uptake. Despite the enhancing effect of the SCFAs on GSH levels, they did not prevent a decrease in glucose uptake which was caused by menadione-induced oxidative stress, suggesting that increases in GSH levels are activated in parallel to the insulin dependent signaling pathways. This hypothesis needs further investigation and should be confirmed in clinical studies.

## Figures and Tables

**Table 1:** Composition of mixtures of SCFAs.

		Acetate		Propionate		Butyrate	
	Concentration Total SCFA (mM)	Relative Amount (%)	Concentration (mM)	Relative Amount (%)	Concentration (mM)	Relative Amount (%)	Concentration (mM)
	1	60	0.6	20	0.2	20	0.2
Mixture 1	5	60	3	20	1	20	1
	20	60	12	20	4	20	4
	1	80	0.8	10	0.1	10	0.1
Mixture 2	5	80	4	10	0.5	10	0.5
	20	80	16	10	2	10	2

## Data Availability

The datasets used and/or analyzed during the current study are available from the corresponding author on reasonable request.
